# Long-term survival of two patients with inoperable post-irradiation osteosarcoma treated with carbon-ion radiotherapy: a case report

**DOI:** 10.1186/s13014-022-02040-3

**Published:** 2022-04-04

**Authors:** Shintaro Shiba, Masahiko Okamoto, Takashi Yanagawa, Isaku Kohama, Kei Shibuya, Shohei Okazaki, Yuhei Miyasaka, Hirotaka Chikuda, Tatsuya Ohno

**Affiliations:** 1 Department of Radiation Oncology, Gunma University Graduate School of Medicine, 3-39-22 Showa-machi, Maebashi, Gunma 371-8511 Japan; 2 Gunma University Heavy Ion Medical Center, Maebashi, Gunma Japan; 3 Department of Orthopaedic Surgery, Gunma University Graduate School of Medicine, Maebashi, Gunma Japan

**Keywords:** Carbon ion radiotherapy, Radiotherapy, Post-irradiation sarcoma, Osteosarcoma, Inoperable sarcoma

## Abstract

**Background:**

Curative treatment of inoperable post-irradiation sarcoma is often challenging, especially using radiotherapy, wherein curative dose administration is difficult because the organs around the tumor have already been irradiated during the first cancer treatment. Carbon-ion radiotherapy (C-ion RT) might be useful in the treatment of post-irradiation sarcomas because it allows re-irradiation with high-dose localization properties and also demonstrates higher cytotoxic effects on radioresistant tumors compared with X-rays. This study presents the long-term survival of two patients with inoperable post-irradiation pelvic osteosarcoma treated with C-ion RT after uterine cervical cancer treatment.

**Case presentation:**

The durations from prior radiotherapy to the diagnosis of post-irradiation osteosarcoma were 112.8 and 172.2 months, respectively. Both patients received 70.4 Gy (relative biological effectiveness) in 16 fractions of C-ion RT, and chemotherapy was performed before and after C-ion RT. Both patients achieved a complete response 1 year after the initiation of C-ion RT. However, one patient developed single lung metastasis 12.6 months after the initiation of C-ion RT and underwent thoracoscopic lobectomy. After 63.7 and 89.0 months from the initiation of C-ion RT, respectively, the patients were alive with no evidence of local recurrence, other distant metastasis, or fatal toxicities.

**Conclusions:**

The study findings suggest that C-ion RT is a suitable treatment option for inoperable post-irradiation osteosarcoma.

## Background

Radiotherapy (RT) is widely known to be an oncologic risk factor, and post-irradiation sarcomas can develop in patients who receive RT for another malignancy [1, 2]. In 1948, Cahan et al. defined the criteria for the diagnosis of post-irradiation sarcomas as follows: (1) history of RT, (2) asymptomatic latent period of several years, (3) development of sarcoma within a previous RT field, and (4) histological confirmation of the sarcomatous nature of the post-RT lesion [3]. These criteria have since been used for the diagnosis of post-irradiation sarcomas [4].

Uterine cervical cancer is expected to be highly curable with RT, and many patients survive for a long time after treatment [5]. However, post-irradiation sarcoma is sometimes a problem in these patients. The incidence of post-irradiation sarcomas after RT for uterine cervical cancer is 0.6%, representing a 22.0-fold increased risk of developing post-irradiation sarcomas compared with the general population [2]. Patients with clinically inoperable post-irradiation sarcomas have limited treatment options [6]. Although RT is an option for such patients, it may be difficult to administer a curative dose because the tolerable dose to the healthy surrounding normal organs may have been exceeded due to the first irradiation. Additionally, post-irradiation sarcomas are considered radioresistant, and local control by RT may be difficult.

Carbon-ion (C-ion) RT might be useful for post-irradiation sarcomas because it allows re-irradiation with high dose localization properties due to the distal tail-off by the Bragg peak and sharp lateral penumbra [7, 8]. Additionally, it shows a higher cytotoxic effect than X-rays on radioresistant tumors, such as sarcomas, due to a higher linear energy transfer [9–11]. With these advantages, post-irradiation sarcomas that are usually difficult to cure with conventional RT could be cured with C-ion RT. Here, we present two patients who underwent C-ion RT for inoperable post-irradiation osteosarcoma after uterine cervical cancer treatment.

## Case presentation

### Patients and treatment

**Fig. 1:**
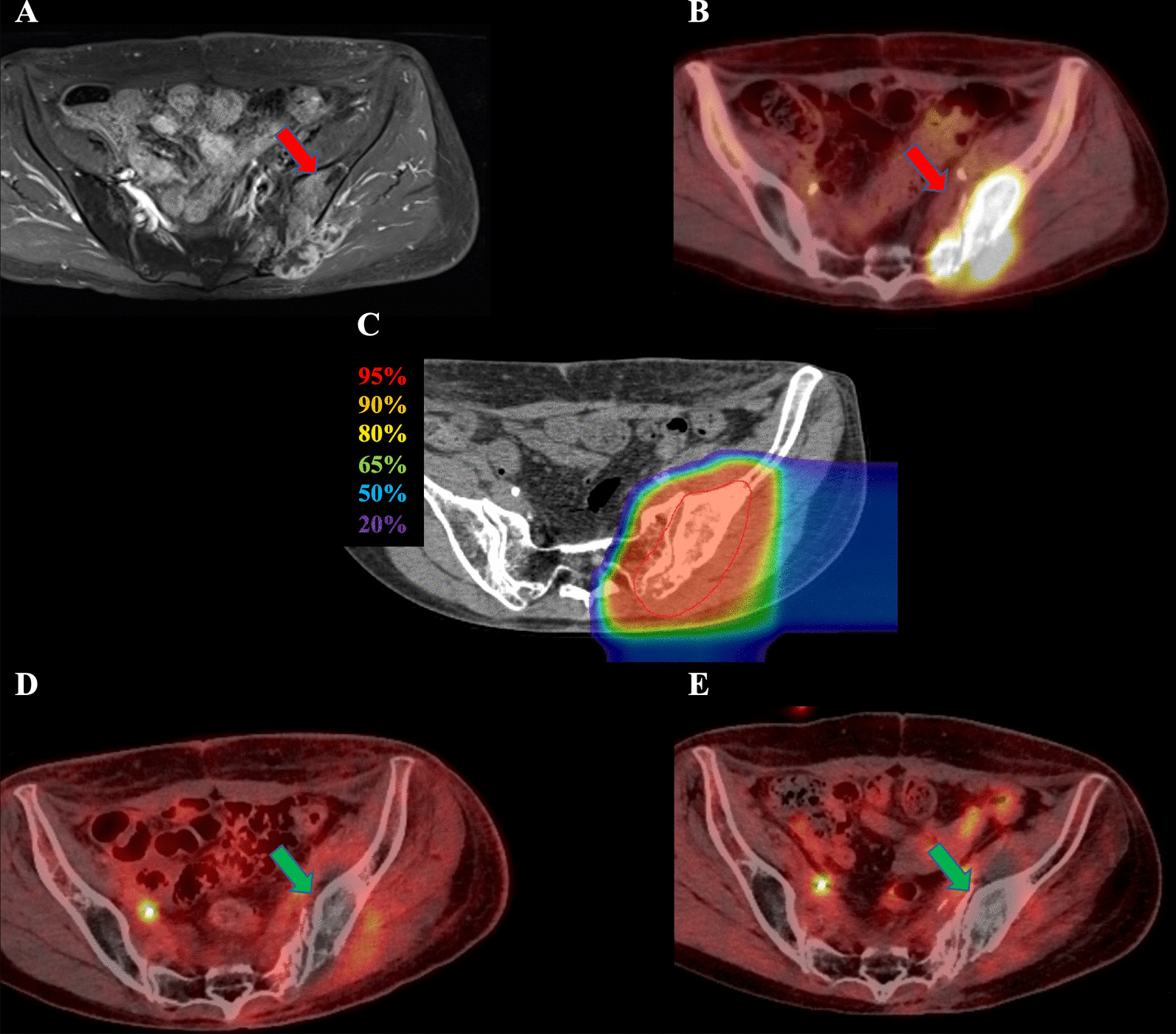
Radiological images before and after carbon-ion radiotherapy (C-ion RT) and dose distribution of Case 1. **a** Contrast-enhanced magnetic resonance imaging (MRI) before C-ion RT. The tumor (50 × 80 × 95 mm) was located in the left iliac bone and had good contrast enhancement (red arrow). **b** 2-deoxy-2-[^18^F]fluoro-D-glucose-positron emission tomography (FDG-PET) before C-ion RT. The red arrow shows the tumor with abnormal FDG uptake. **c** Dose distribution on axial computed tomography images. The area within the red outline is the gross tumor volume of the osteosarcoma. The 95% (red), 90% (orange), 80% (yellow), 65% (green), 50% (blue), and 20% (purple) isodose curves are highlighted (100% = 70.4 Gy [relative biological effectiveness]). **d** FDG-PET 1 year after C-ion RT. FDG uptake was decreased compared to that before treatment (green arrow). (E) FDG-PET 5 years after C-ion RT. FDG uptake was decreased compared to that before treatment (green arrow)

**Fig. 2:**
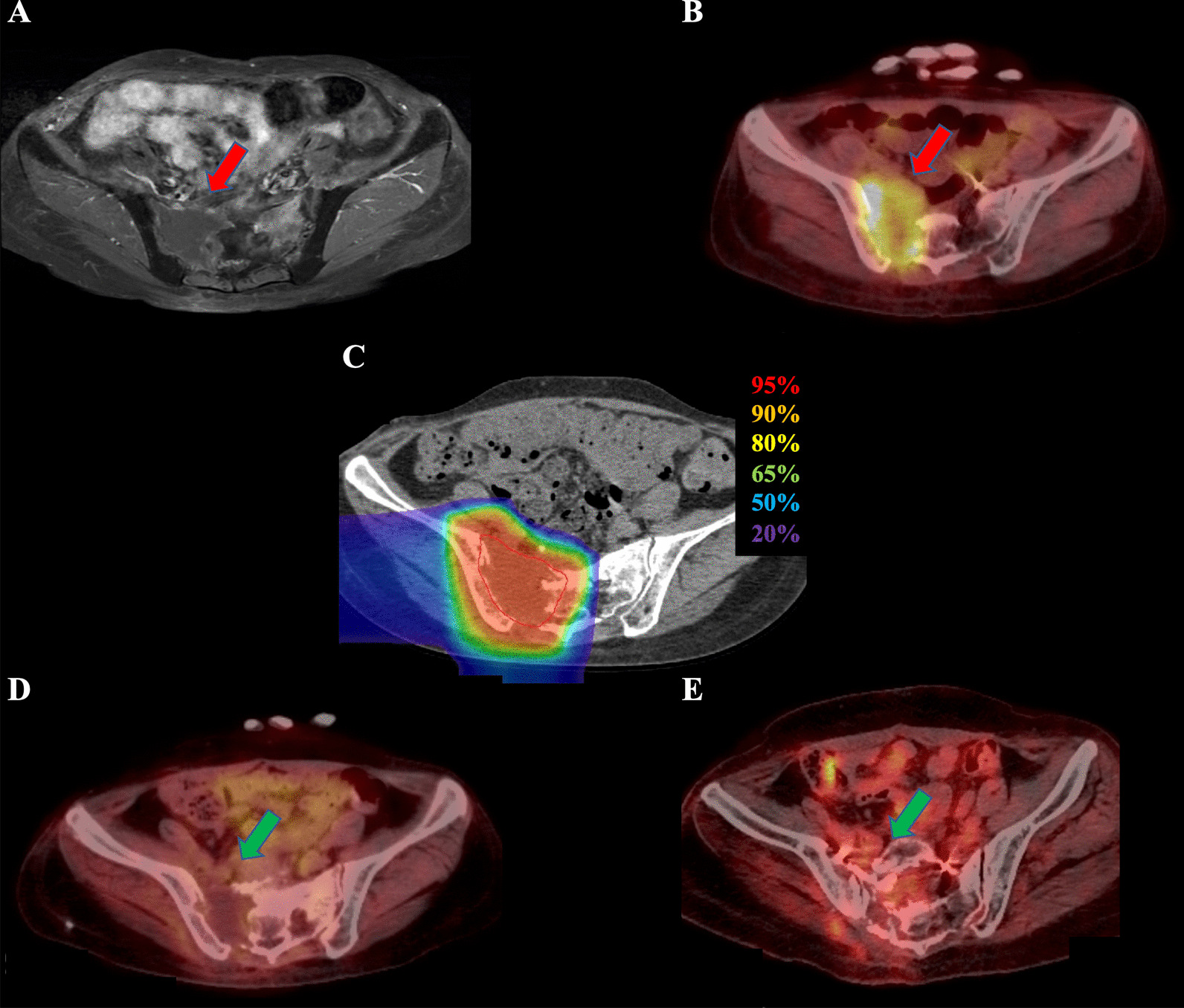
Radiological images before and after carbon-ion radiotherapy (C-ion RT) and dose distribution of Case 2. **a** Contrast-enhanced magnetic resonance imaging (MRI) before C-ion RT. The tumor (51 × 52 × 68 mm) was located in the sacral bone and had good contrast enhancement (red arrow). **b** 2-deoxy-2-[^18^F]fluoro-D-glucose-positron emission tomography (FDG-PET) before C-ion RT. The red arrow shows the tumor with abnormal FDG uptake. **c** Dose distribution on axial computed tomography images. The area within the red outline is the gross tumor volume of the osteosarcoma. The 95% (red), 90% (orange), 80% (yellow), 65% (green), 50% (blue), and 20% (purple) isodose curves are highlighted (100% was 70.4 Gy [relative biological effectiveness]). **d** FDG-PET 1 year after C-ion RT. FDG uptake was decreased compared with that before treatment (green arrow). **e** FDG-PET 7 years after C-ion RT. FDG uptake was decreased compared with that before treatment (green arrow)

Two patients with inoperable post-irradiation pelvic osteosarcoma after uterine cervical cancer treatment were referred to our department; both received postoperative RT without chemotherapy for uterine cervical cancer. The durations from prior RT to the diagnosis of post-irradiation osteosarcoma were 112.8 and 172.2 months, respectively. Both patients underwent magnetic resonance imaging, contrast-enhanced computed tomography, and 2-deoxy-2-[^18^F]fluoro-D-glucose-positron emission tomography (FDG-PET). Figures 1 and 2 show diagnostic imaging results before C-ion RT. Both patients had locally advanced disease, were unsuitable for surgery, and had no distant metastases or direct infiltration to the gastrointestinal tract. The patient characteristics and treatments are summarized in Table 1. The eighth edition of the Union for International Cancer Control/American Joint Committee on Cancer TNM staging system was used for tumor staging [12].

**Table 1:** Patient characteristics and treatment

	Case 1	Case 2
*Treatment for uterine cervical cancer*		
Purpose	Postoperative RT	Postoperative RT
Dose of prior RT	EBRT 50 Gy/25 fr.*	EBRT 50 Gy/25 fr.*
	ICBT 7 Gy/1 fr	ICBT 24 Gy/4 fr
Chemotherapy for uterine cervical cancer	None	None
*C-ion RT for post-irradiation osteosarcoma*		
Duration from prior RT to diagnosis of post-irradiation osteosarcoma, months	112.8	172.2
Age at registration of C-ion RT years	67	55
Tumor location	Left iliac bone	Sacral bone
Tumor size, mm	50 × 80 × 95	51 × 52 × 68
Staging	cT2bN0M0	cT2bN0M0
Histological analysis	Conventional osteosarcoma	Conventional osteosarcoma
Chemotherapy before C-ion RT	Methotrexate and vincristine	Methotrexate
	Doxorubicin and cisplatin	Pirarubicin and cisplatin
	Pazopanib	
Dose of C-ion RT	70.4 Gy (RBE)/16 fr	70.4 Gy (RBE)/16 fr
Chemotherapy after C-ion RT	Pazopanib	Pazopanib
		Methotrexate

C-ion RT, carbon-ion radiotherapy; EBRT, external-beam radiotherapy; fr, fractions; ICBT, intracavitary brachytherapy; RBE, relative biological effectiveness; RT, radiotherapy

^*^The last 20 Gy was delivered using a central shielding technique

Both patients received 70.4 Gy (relative biological effectiveness [RBE]) in 16 fractions for 4 weeks. The microdosimetric kinetic model was used to calculate the RBE, and doses of C-ion RT were expressed as RBE-weighted dose [Gy (RBE)], which was defined as the physical dose multiplied by the RBE of the C-ions [13]. C-ion RT was performed using passive scattering irradiation, with the beams in one direction per fraction. The patients received C-ion RT once daily for 4 days a week (Tuesday to Friday). Figures 1c and 2c show the dose distribution of C-ion RT. The tumor response was assessed using the Response Evaluation Criteria in Solid Tumors (version 1.1) and FDG-PET [14, 15]. Toxicities were assessed using the Common Terminology Criteria for Adverse Effects (version 4.0) [16].

### Case 1

One year after C-ion RT, a complete metabolic response was observed on FDG-PET (Fig. 1d). However, the patient developed a single lung metastasis 12.6 months after C-ion RT initiation and underwent thoracoscopic lobectomy. The patient is alive 63.7 months after C-ion RT initiation with no evidence of local recurrence, other distant metastasis, or grade 3 or higher toxicities.

### Case 2

One year after C-ion RT, a complete metabolic response was observed on FDG-PET (Fig. 2d). The patient is alive 89 months after C-ion RT initiation with no evidence of local recurrence or distant metastasis. The patient developed grade 3 sacral bone fracture where the sarcoma was located, grade 3 edema of the lower extremities associated with sacral bone fracture, and grade 2 peripheral neuropathy requiring high-dose opioids.

## Discussion and conclusions

The two patients experienced favorable clinical outcomes after C-ion RT for inoperable post-irradiation pelvic osteosarcoma that arose after uterine cervical cancer treatment. These results suggest that C-ion RT, which has a high dose concentration and higher cell-killing effect than other RT modalities, exerts a safe and favorable local effect, even as the second irradiation and for radioresistant tumors, and contributes to long-term survival.

There have been several reports of initial treatment with C-ion RT and proton beam therapy with C-ion RT boost for sarcomas in patients with no history of irradiation which showed favorable clinical outcomes despite including patients with inoperable tumors; the median survival time was 31.2–49.4 months, indicating that some patients had long-term survival [9–11, 17]. The survival times of our patients were longer than this range, despite both patients having previously received irradiation to the pelvis. In a previous report of post-irradiation sarcoma, the median survival was only 37 months, including patients who underwent curative surgery. For patients with inoperable tumors, survival was significantly poorer (median survival: 15 months) [18]. One reason for poor survival in patients with inoperable tumors is that curative irradiation is not possible for post-irradiation sarcomas because of the risks associated with re-irradiation. However, the high dose localization property of C-ion RT enables a curative dose administration with a smaller risk to the surrounding organs, and higher cell-killing effect of C-ion RT provides local control of radioresistant post-irradiation osteosarcoma. Our two patients achieved long-term survival. Therefore, we believe that C-ion RT might be a curative treatment option for inoperable post-irradiation osteosarcoma.

Generally, dose constraints for the gastrointestinal tract in re-irradiated patients are stricter than those at the time of initial irradiation. However, there are no data on these dose constraints or on the recovery of normal tissues in the period between the first and second irradiations. We designed our treatment plan to reduce the gastrointestinal tract dose as much as possible. The total maximum dose to the gastrointestinal tract in the worst-case calculation could have exceeded 100 Gy (RBE) due to overlap with the gastrointestinal tract within the irradiation area of the previous RT and C-ion RT. However, we considered that the dose to the gastrointestinal tract was tolerable because of the long period between the previous RT and C-ion RT and the small high-dose volume in the gastrointestinal tract. Neither patient developed gastrointestinal toxicities.

C-ion RT for head and neck sarcomas, including post-irradiation sarcomas, was previously reported [19]. However, this report included a small number of patients, including those with locally recurrent sarcoma after surgery without RT, and those who received a combination of proton-beam therapy and C-ion RT. Therefore, this cannot be considered a coherent report of C-ion RT for post-irradiation sarcomas. To our knowledge, this is the first report of C-ion RT for post-irradiation osteosarcoma.

A limitation of this report is that it is not an analysis to confirm the safety and efficacy of C-ion RT for post-irradiation sarcomas. Further investigation with a larger sample size is warranted to establish the safety and efficacy of C-ion RT.

In conclusion, the study findings suggest that C-ion RT is a potential treatment option for inoperable post-irradiation osteosarcoma.

## Data Availability

The datasets generated for this report are available from the corresponding author on reasonable request..

## Abbreviations

RT: Radiotherapy
C-ion: Carbon-ion
FDG-PET: 2-Deoxy-2-[^18^F]fluoro-D-glucose-positron emission tomography
RBE: Relative biological effectiveness
